# The poly-proline motifs found in the cytoplasmic tail of the brush border cadherin CDHR5 play a role in its ability to elongate microvilli

**DOI:** 10.17912/micropub.biology.001663

**Published:** 2025-08-22

**Authors:** Samaneh Matoo, Prashun Acharya, Sadika T.J. Tonu, Jasvinder Bharaj, Ashwini Mudaliyar, Basmala Touny, Regan VanderPol, Morgan Timms, Nicole Dyko, Domtie Asante, Hawa Abdulle, Scott W. Crawley

**Affiliations:** 1 Molecular, Cellular and Developmental Biology, University of Toledo, Toledo, Ohio, United States

## Abstract

Cadherin-related family member 5 (CDHR5) is a protocadherin found enriched at the tips of brush border microvilli of the gut and kidney, where it plays an important role in the development of these specialized microvilli. CDHR5 is a type-1 transmembrane protein with a short cytoplasmic tail that contains a number of poly-proline motifs of unknown function. We performed an analysis of the poly-proline stretches in the CDHR5 cytoplasmic tail and show that mutation of these motifs does not largely influence the targeting of CDHR5 to microvilli, but does significantly impact the ability of the cadherin to promote microvillar elongation.

**Figure 1. Analysis of the poly-proline motifs in CDHR5:**
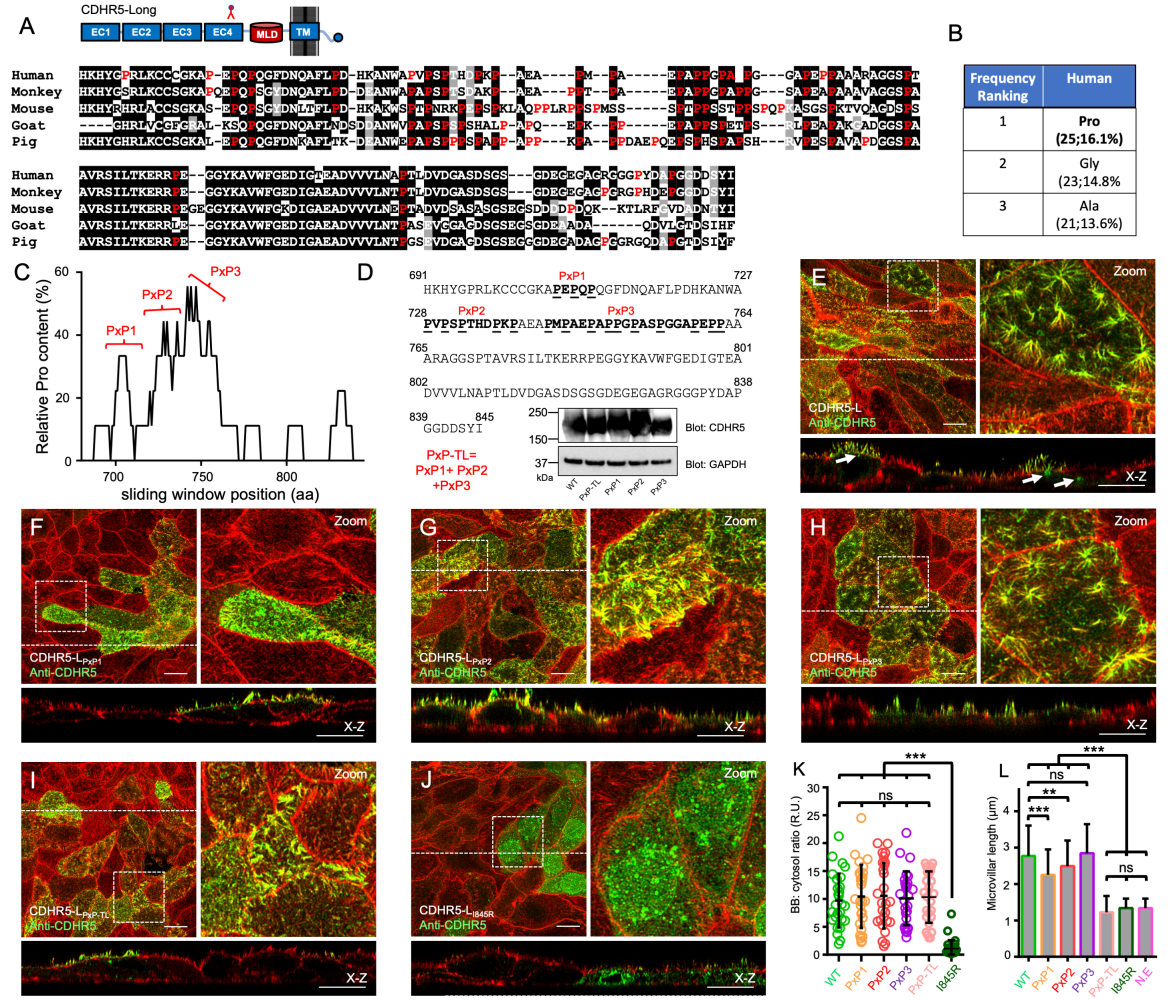
(A) Sequence alignment of the cytoplasmic tail of CDHR5 from Human (Homo sapiens), Monkey (Macaca mulatta), Mouse (Mus musculus), Goat (Capra hircus), and Pig (Sus scrofa). Amino acids shaded in black are identical. Amino acids shaded in gray are similar. Amino acids colored in red show proline residues. Shown above the alignment is a cartoon diagram of CDHR5 that indicates where the anti-human CDHR5 antibody binds. (B) Table of the three most highly enriched amino acids in the CDHR5 cytoplasmic tail. Following the amino acid identity is the number of occurrences of the amino acid within the tail and the % composition. (C) Analysis of the relative proline content of the CDHR5 cytoplasmic tail, which identifies three proline clusters that were targeted for mutagenesis. (D) Sequence of the human CDHR5 cytoplasmic tail showing the PxP clusters and their sequences after mutation. The PxP-TL mutant combines all three mutant clusters together. Also shown are immunoblots for CDHR5 and GAPDH from total cell lysates of the CDHR5 WT and PxP-mutant stable cell lines. GAPDH was used as a loading control. (E-J). Confocal images of a 4-day polarized LLC-PK1-CL4 monolayer expressing WT, PxP mutants and the I845R PBM mutant CDHR5 visualized for CDHR5 (green) and F-actin (red). The boxed regions denote the area in the zoomed panels. The dashed line indicates the position where the x–z sections were taken; the x–z sections are shown below the *en face* image. Arrows in the *x-z* section of WT CDHR5 point to examples of cytoplasmic puncta. Scale bars, 10 μm. (K) Scatterplot quantification of the BB:cytosol ratios of CDHR5 signal in all constructs tested. Bars indicate mean and standard deviation. R.U = relative units. Measurements: 27 BB:cytosol measurements from independent cells for each construct were collected from stable cell lines that were derived twice independently. ns=not significant ***p < 0.0001, two-tailed t test. (L) Quantification of microvillar length from cells overexpressing WT or mutant CDHR5 constructs compared to non-expressing (N.E) cells. Bars indicate mean ± standard deviation. Measurements: CDHR5 WT expressing, N=180 microvilli, CDHR5-PxP1 expressing, N=117 microvilli, CDHR5-PxP2 expressing, N=119 microvilli, CDHR5-PxP3 expressing, N=118 microvilli, CDHR5-PxP-TL expressing, N=119 microvilli, CDHR5-I845R expressing, N=83 microvilli, non-expressing cells N=83 microvilli. ***p < 0.0001, **p < 0.001, ns=not significant, two-tailed t test.

## Description

The transporting epithelial cells that line the gut and proximal convoluted tubule of the kidney build a dense collection of microvilli on their apical surface known as a brush border (BB) (Crawley et al., 2014a). Formation of BB microvilli serves as a mechanism to amplify the amount of apical membrane surface area of an epithelial cell exposed to the luminal environment of their organ, in order to maximize solute transport. Proper BB formation requires a protocadherin-based adhesion complex found at the tips of microvilli known as the intermicrovillar adhesion complex (IMAC)(Choi et al., 2020; Crawley et al., 2014b; Crawley et al., 2016; Graves et al., 2020). During BB development, the IMAC creates physical protein-based linkages known as “intermicrovillar adhesion links” that connect neighboring microvilli together at their tips, driving their assembly into a uniform ordered BB. Intermicrovillar adhesion links are formed by a trans-heterophilic interaction of two protocadherins, CDHR2 and CDHR5. Loss of either of these cadherins in mice results in BB defects in enterocytes that include having a lower density of apical microvilli that are shorter in length (Modl et al., 2023; Pinette et al., 2019). Consistent with this, overexpression of CDHR5 in epithelial cells drives pronounced lengthening of BB microvilli (Matoo et al., 2024). This effect is dependent upon the correct trafficking of CDHR5 to the apical domain, which is influenced by its cytoplasmic tail (Matoo et al., 2024). All known splice isoforms of CDHR5 have a common cytoplasmic tail, and only differ in presence and composition of a mucin-like domain (MLD) in their ectodomain (Goldberg et al., 2000; Goldberg et al., 2002). The cytoplasmic tail of CDHR5 is ~155 residues long and contains a number of potential poly-proline motifs along with a C-terminal PDZ binding motif (PBM)(Goldberg et al., 2000). Importantly, CDHR5 splice isoforms that contain an MLD in their ectodomain require an intact C-terminal PBM in their tail in order to traffic correctly to apical microvilli to promote microvillar elongation (Matoo et al., 2024). It is presently unclear, however, whether the poly-proline stretches found in the CDHR5 tail play any role in its biology. While a yeast-two-hybrid screen of the CDHR5 cytoplasmic tail against a kidney cDNA library identified the PDZ-based scaffolds E3KARP/EBP50 as binding partners for the PBM of CDHR5, no SH3-containing proteins were discovered in the screen that could be potential interactors for the poly-proline motifs (Matoo et al., 2024). In this study, we sought to determine whether the poly-proline motifs of CDHR5 were necessary for its delivery to apical microvilli or its ability to promote microvillar elongation.

For our experiments here, we used the canonical or “long” isoform of CDHR5 which contains the largest predicted MLD variant in its ectodomain of all known splice isoforms of the cadherin ( Fig. 1A ; top panel cartoon). Our previous immunoblot analysis suggests this is the most highly expressed isoform of CDHR5 in enterocytes (Matoo et al., 2024). We first performed a sequence analysis of the cytoplasmic tail of CDHR5 across a number of mammalian species and found that in each case, proline was the most highly enriched amino acid in the tail. For human CDHR5, there are 25 proline residues in its tail, which constitutes ~16% of the total amino acid content of the tail ( Fig. 1A, B). To analyze the relative distribution of proline residues across the human CDHR5 cytoplasmic tail, we used a 20-residue sliding window algorithm to scan along the tail sequence to detect regions enriched in this residue. We found that the proline content of the tail could be roughly broken down into three clusters ( Fig. 1C ). We designated each cluster with a name (PxP1, PxP2 and PxP3) and created mutant versions in which all proline residues within each specific cluster were changed to alanine ( Fig. 1D ). We further created a version of CDHR5 that had all three proline clusters mutated to alanine, which we designed PxP-TL. Finally, we also utilized a mutant of CDHR5 that had the C-terminal PBM ablated (I845R) which served as a low-targeting control based on our previous study (Matoo et al., 2024). This mutation blocks the ability of CDHR5 to interact with protein scaffolds E3KARP/EBP50 that are highly enriched in apical microvilli of transporting epithelial cells (Ingraffea et al., 2002).

Each PxP mutant construct along with wild-type (WT) and I845R CDHR5 were incorporated into the pLVX-PURO lentiviral vector for the production of lentiviral particles. This vector generates an untagged version of CDHR5, leaving the C-terminal carboxyl group of the CDHR5 PBM intact which is necessary for its binding interactions and overall function. Blocking the C-terminal carboxyl group of the CDHR5 PBM with a GFP moiety, for example, prevents correct apical targeting of the long isoform of CDHR5 (Matoo et al., 2024). The resulting lentivirus was used to transduce LLC-PK1-CL4 cells to generate stable cell lines expressing each construct. LLC-PK1-CL4 cells are a pig proximal convoluted tubule kidney cell line that rapidly polarize in culture to generate easily-visualized apical microvilli. Immunoblot analysis of these PXP-mutant stable cell lines using an antibody directed against the fourth extracellular cadherin (EC) domain in the ectodomain of human CDHR5 ( Fig. 1A ; see cartoon panel), showed that each mutant was expressed at a level similar to WT CDHR5 ( Fig. 1D ). Importantly, this antibody does not cross-react with pig CDHR5, allowing us to specifically detect our transduced human constructs only (Matoo et al., 2024). Stable cell lines were then plated on coverslips and allowed to polarize for 3 days, after which cells were fixed and stained for F-actin using Phalloidin Alexa Fluor-568 and CDHR5 using our antibody.

Confocal imaging of the WT and poly-proline mutant samples revealed that the majority of the CDHR5 signal was found in apical microvilli ( Fig. 1E- I). As we observed previously for this splice isoform of CDHR5 that contains an MLD, we did note that a fraction of the expressed cadherin was found trapped internally as puncta of varying sizes (see arrows in X-Z section of Fig. 1E that point to examples of puncta). To measure the efficiency of targeting for each CDHR5 construct, we quantified the distribution of CDHR5 signal found in the BB versus the cytosol. Identical to WT protein, we observed that there was an approximate ten-fold enrichment of the CDHR5 poly-proline mutants found in microvilli versus the cytoplasm ( Fig. 1K ). In stark contrast, disrupting the C-terminal PBM of CDHR5 using the I845R mutation largely blocked the ability of the cadherin to target to apical microvilli in appreciable amounts ( Fig. 1J- K). Finally, we tested whether disrupting the poly-proline clusters in CDHR5 had any effect on the ability of the cadherin to promote the elongation of microvilli once correctly delivered to the apical domain. Measuring the length of microvilli in X-Z cross sections revealed that mutating PxP1 and PxP2 impacted the ability of CDHR5 to promote microvillar elongation, while mutating PxP3 had no effect. Interestingly, mutating all three PxP clusters appeared to have an additive effect in which the overexpressed cadherin no longer induced microvillar elongation ( Fig. 1L ). Indeed, the length of microvilli measured in the PxP-TL construct was not significantly different compared to the CDHR5 I845R PBM mutant or the length of microvilli measured for cells that were not over-expressing human CDHR5 (non-expressing-N.E cells). All together, these results demonstrate the poly-proline clusters found in the CDHR5 cytoplasmic tail do not play a direct role in its apical targeting in kidney cells, but do contribute to the ability of the cadherin to promote microvillar elongation. These finding may also directly translate to other tissue systems, such as the gut, since CDHR5 has been found to regulate the length of intestinal microvilli (Modl et al., 2023). Future investigations will be necessary to understand how the CDHR5 poly-proline clusters are involved in promoting microvillar elongation.

## Methods

**Molecular Biology**

The human cDNA construct of the canonical or long isoform of CDHR5 (NM_021924.5;UniProtKB-Q9HBB8-1) was cloned into the pCR8-GW-TOPO entry vector. PxP mutants were generated by synthesizing mutant gene fragments encoding the cytoplasmic tail of CDHR5, which were then cloned into the full-length gene using available restriction enzyme sites that flanked the mutated region. The CDHR5 I845R PBM mutant was generated from the parental pCR8-GW-CDHR5 construct using the Quikchange site-directed mutagenesis kit (Aligent) following the manufacturers’ protocol. Entry clones of all constructs were shuttled into the pLVX-PURO vector (Takara) that had been Gateway-adapted using the Gateway Vector Conversion kit (Invitrogen).

**Cell culture and stable cell line generation**

LLC-PK1-CL4 and HEK293FT cells were grown in a 5% CO2 humidified incubator at 37°C in DMEM supplemented with high glucose, 2 mM L-glutamine and 10% FBS. HEK293FT cells (10 cm dish at 80% confluency) were used for lentivirus production by co-transfecting 6 μg of Lentiviral expression plasmid with 4 μg psPAX2 packaging plasmid and 0.8 μg pMD2.G envelope plasmid using the polyethylenimine transfection reagent (Polysciences). Cells with transfection medium were exchanged with fresh medium 12 hours after transfection. Cells were returned to the 37°C incubator with 5% CO2 to allow for lentiviral production into the medium. Two days later, supernatant containing secreted lentiviral particles was collected, filtered with a 0.45 μm syringe filter and concentrated using Lenti-X Concentrator (Takara) and stored at -80°C for further use. To transduce LLC-PK1-CL4 with lentivirus, cells were seeded and grown to 80% confluency in a T25 flask, after which the medium was supplemented with 8 μg/ml polybrene and ~300μl of concentrated lentivirus was added. Cells were then incubated overnight with this transduction medium. Fresh medium was then swapped in and cells were allowed to recover for 24 hours. To select for stable cell lines after viral transduction or plasmid transfection, T25 flasks containing the transduced/transfected cells were reseeded to 10 cm dishes and grown for 3 days in the absence of antibiotics. Next, cells were reseeded into T182 flasks with medium containing 50 μg/ml of puromycin. Cells were then continuously grown for numerous (~10) passages to select for stable integration of DNA.

**Confocal Microscopy**

LLC-PK1-CL4 cells seeded on coverslips were allowed to polarize for 4 days. After polarization, cells were fixed in 4% paraformaldehyde (Electron Microscopy Sciences) in PBS for 15 min at RT, washed with PBS, and permeabilized with 0.1% Triton X-100 in PBS for 7 min. After fixation, cells were washed four times with PBS and blocked overnight with 5% BSA at 4°C. Cells were stained for 1 hr at room temperature using primary antibody for anti-CDHR5. Cells were then washed three times with PBS and incubated with Alexa Fluor 488 goat anti-rabbit and Alexa Fluor 568 phalloidin diluted 1:200 in PBS for 1 hr at RT. Coverslips were then washed and mounted on slides with ProLong Diamond for imaging. Imaging was done using a Leica TCS SP8 laser-scanning confocal microscope equipped with HyVolution deconvolution software.

**Image and Statistical analysis**

Image analysis was performed using ImageJ2 Version 2.3.0 (National Institutes of Health). For analysis of construct enrichment in microvilli, we calculated the ratio of BB to cytosolic signal intensity as previously described (Graves et al., 2020; Matoo et al., 2024). The data were analyzed in a blinded fashion. All graphs were generated and statistical analyses performed using Prism version 6 (GraphPad). For all figures, error bars represent S.D. Unpaired t tests were employed to determine statistical significance between reported values.

## Reagents

**Molecular Biology reagents:**

Quikchange site-directed mutagenesis kit (Aligent catalog no. 200519)

LR Clonase II (Invitrogen catalog no. 11791100)

Gateway Vector Conversion System with One Shot ccdB Survival Cells (Invitrogen catalog no. 11828029)

Gene synthesis performed by Twist Biosciences

**Cell culture reagents:**

DMEM high glucose, L-glutamine, sodium pyruvate (Sigma-Aldrich catalog no. D6429)

Fetal Bovine Serum, Qualified (Gibco catalog no.10437-028)

Polyethylenimine transfection reagent PEI-MAX (Polysciences catalog no. 24765)

Lenti-X Concentrator (Takara catalog no. 631232)

**Microscopy and Immunoblot reagents:**

Anti-CDHR5 (1:200; Sigma catalog no. HPA009081)

Phalloidin-Alex568 (1:300; Invitrogen catalog no. A12380)

Goat Anti-rabbit Alex488 (1:200; Invitrogen catalog no. A-11008)

ProLong Diamond Antifade Mount (Invitrogen catalog no. P36970)

Anti-GAPDH (1:10,000; Protein Tech catalog no. 60004-1-Ig)

Goat anti-Mouse Secondary HRP (1:10,000 catalog no. 31430)

Goat anti-Rabbit Secondary HRP (1:10,000 catalog no. 31460)
